# HER2 status in gastro-oesophageal adenocarcinomas assessed by two rabbit monoclonal antibodies (SP3 and 4B5) and two *in situ* hybridization methods (FISH and SISH)

**DOI:** 10.1111/j.1365-2559.2011.03760.x

**Published:** 2011-02

**Authors:** James E Boers, Harriëtte Meeuwissen, Natalie Methorst

**Affiliations:** Pathology, Isala kliniekenZwolle, the Netherlands

**Keywords:** Anti-HER2 therapy, gastric adenocarcinoma, HER2, immunohistochemistry, *in situ* hybridization, oesophageal adenocarcinoma

## Abstract

**Aims:**

HER2 gene amplification has been detected in 10–20% of gastric adenocarcinomas. In view of the recently demonstrated clinical benefit of the anti-human epidermal growth factor receptor 2 (HER2) drug trastuzumab in the treatment of advanced gastric cancer, reliable HER2 testing is of key importance. The aim of this study was to examine HER2 status in gastro-oesophageal adenocarcinomas comparing SP3 and 4B5 immunohistochemistry (IHC) with dual probe HER2 [fluorescence *in situ* hybridization (FISH) and silver *in situ* hybridization (SISH)].

**Methods and results:**

IHC and SISH were carried out on biopsy specimens of 146 patients with adenocarcinomas of the oesophagus and stomach. All SP3-IHC-positive cases and 91% of 4B5-IHC-positive cases were amplified. Sensitivity of SP3-IHC-positivity and 4B5-IHC-positivity for amplification was 77% and 96%, respectively. Results of FISH performed in 42 cases were identical to SISH. Amplification was heterogeneous in 73% of the adenocarcinomas; 24% of the oesophago-gastric carcinomas and 7% of distal stomach tumours were amplified.

**Conclusions:**

HER2-positivity is present in a significant proportion of oesophago-gastric adenocarcinomas (24%), but at a lower rate in the distal stomach (7%). Sensitivity for amplification is higher with 4B5 IHC than with SP3. FISH and SISH yield identical results, but assessment is much easier with SISH. Our findings provide important guidance for HER2-testing in gastro-oesophageal adenocarcinomas for patients in whom anti-HER2 treatment is considered.

## Introduction

Therapies directed against tumours overexpressing the transmembranous human epidermal growth factor receptor 2 (HER2) as a result of HER2-amplification has become widely available in the last decade for breast carcinomas. HER2-positivity is reported in other carcinomas, most notably gastric and oesophageal adenocarcinomas.^1^–^19^ A large phase III trial employing trastuzumab, directed against the HER2 protein, has been conducted for advanced gastric carcinomas showing clinical benefit (ToGA trial^20^).

Adenocarcinomas of the distal oesophagus, oesophago–gastric junction (EGJ) and gastric cardia carcinomas share many risk factors, and the incidence of these tumours has risen dramatically in the developed world.^21^,^22^ On the other hand, gastric carcinomas situated in the body or antrum are epidemiologically and biologically distinct from adenocarcinomas situated at or near the EGJ. While the incidence of distal gastric carcinomas is decreasing in industrialized countries, they still constitute a major global health problem. A significant proportion of patients with distal oesophageal or gastric carcinomas presents in an advanced disease stage resulting in poor overall survival.^23^,^24^ A first trial with anti-HER2 therapy in advanced gastric adenocarcinoma showed clinical benefit, and with other ongoing trials in advanced gastric and oesophageal adenocarcinomas, reliable HER2 status assessment in both oesophageal and gastric adenocarcinomas is likely to become increasingly important.

HER2 status is usually determined by immunohistochemistry (IHC) and/or *in situ* hybridization (ISH). With IHC the four-tiered scoring system described originally for the Food and Drug Administration (FDA)-approved HercepTest™ (Dako, Glostrup, Denmark) is used widely, irrespective of the IHC method employed. Samples scored as 0 and 1+ are negative, 2+ as equivocal and 3+ as positive. In the original algorithm for breast cancer, only cases with 2+ score had to be retested with ISH. However, American Society of Clinical Oncology/College of American Pathologists (ASCO/CAP) guidelines^25^ require in-house validation of 1+ and 3+ samples with ISH before a certified laboratory can confine ISH retesting to 2+ samples.

Recently, a modification of the HercepTest™ scoring system for gastric carcinomas was proposed.^3^ The original system required circular staining for a 2+/3+ score and staining of >10% tumour cells in breast cancer. As non-circular basolateral IHC staining was observed frequently in gastric carcinomas, as well as strong (3+) staining of <10% tumour cells in biopsies, these elements were added to the original HercepTest™ system.

Novel rabbit monoclonal HER2 antibodies have been introduced recently claiming higher avidity and lower background staining. The 4B5 antibody (Ventana Medical Systems, Tucson, AZ, USA) is directed against the extracellular domain of the HER2-receptor and is FDA-approved. Another antibody is SP3 (Labvision; Thermo Fisher Scientific, Fremont, CA, USA) directed against the intracellular domain providing clearer staining, but possibly lower sensitivity.^26^ Both antibodies claim an excellent correlation with ISH.^27^,^28^

The PathVysion® FDA-approved fluorescence ISH (FISH; Abbott, Abbott Park, IL, USA) is the classic *in situ* hybridization test using probes for HER2 and chromosome 17 (Chr17) concomitantly on one slide, allowing for the calculation of a HER2:Chr17 ratio. Dako PharmDx™ FISH used for HER2 testing in the ToGA trial^2^,^3^,^20^ uses a similar approach. FISH requires a fluorescence microscope and assessment in biopsies with heterogeneous staining patterns can be extremely laborious. ISH methods allowing traditional transmitted light microscopy have been introduced recently. The dual-probe silver *in situ* hybridization (SISH INFORM®; Ventana) uses two separate slides for the HER2 and Chr17 probes which allows for a computed HER2:Chr17 ratio. Excellent FISH/SISH correlation is claimed.^29^

No results have been published previously using SP3 and/or 4B5 IHC or SISH in gastric or oesophageal adenocarcinomas. We conducted a single institution study in 146 patients using the two antibodies with SISH. In addition, all cases showing 1+ immunoscore or higher were retested with Dako FISH. The objective was to determine the predictive value of both antibodies for and the incidence of HER2-amplification.

## Patients and methods

The study includes biopsy specimens from 178 consecutive patients with the diagnosis of adenocarcinoma of the stomach or distal oesophagus (study period 1999–2007). Sufficient material for immunohistochemistry (IHC) and *in situ* hybridization (ISH) studies was available in 146 cases with formalin-fixed, paraffin-embedded primary tumour biopsies. The average number of biopsies per case was 5.8 [range 2–14, standard deviation (SD) 2.4]. Location of the tumour was noted (distal oesophagus, gastric cardia, body or antrum). Oesophago-gastric region (EGR) was defined as the distal oesophagus, the EGJ or gastric cardia. Distal stomach was defined as gastric body or antrum. On the newly cut slides stained with haematoxylin and eosin (H&E), the tumour was typed as ‘intestinal’, ‘diffuse’, ‘mucinous’ or ‘mixed’ using the Laurén classification.

HER2 IHC studies were carried out on the NeXes stainer (Ventana) using 3-μm slides after antigen retrieval (10 mm citrate, pH 7.3, boiling time 25 min). On all slides, three breast cancer tumour samples with an immunoscore of 0, 1+ and 3+ were used as controls. The rabbit monoclonal antibody SP3 was used at a dilution of 1:20 with a 32-min incubation time at 37°C. Subsequently, iView DAB,3,3′– diaminobenzidine, (Ventana) was carried out for visualization. In our practice, 300 cases of breast cancer specimens were compared with this SP3 protocol in comparison with DAKO FISH (see below for protocol), yielding excellent concordance. Similarly, 4B5 was applied in prediluted form as provided by the manufacturer.

For scoring of HER2 immunoreactivity, the modified Herceptest™ four-tiered scoring system developed for gastric adenocarcinoma by Hofmann *et al.*^3^ was used; scores were: 0 (negative): no staining or membrane staining in <10% of cells; 1+ (negative): faint/barely perceptible membrane reactivity in >10% of cells; cells are only stained in part of their membrane; 2+ (equivocal): weak to moderately complete or basolateral reactivity in >10% of tumour cells; and 3+ (positive): moderate to strong complete or basolateral reactivity of >10% of tumour cells. Cohesive IHC 3+ clones irrespective of the proportion of staining cells, i.e. <10%, are also considered positive, as only biopsy material was examined. The number of biopsies, the number of positive biopsies and HER2-immunoreactivity expressed as a percentage of total tumour present was noted. A histoscore (H-score) was calculated: HER2 H-score = [percentage immunoreactive tumour cells (0–100%)] × [immunoscore (0, 1, 2 or 3)]. Background staining was noted in 4B5 immunostaining; ‘strong’ background was specified as both nuclear and diffuse cytoplasmic staining, and ‘light’ background as diffuse cytoplasmic immunoreactivity only.

SISH was studied in all cases using the INFORM HER2 DNA (Ventana) kit with HER2 and Chr17 probes on separate serial-sectioned 4-μm slides with 8 min and 12 min pepsin pretreatment, respectively. SISH was carried out using the Benchmark XT stainer (Ventana) with an otherwise standardized protocol supplied by the manufacturer.

FISH was carried out with the HER2 FISH PharmDx™ kit (Dako) in 40 cases with SP3 and/or 4B5 HER2-immunoreactivity of 1+ and higher; two cases with only strong background staining in 4B5 immunohistochemistry were added. Three-micrometer slides were deparaffinized, placed in pretreatment solution at 95°C for 10 min, and after 15 min cooling at room temperature (RT) put into wash buffer twice for 3 min. Then, pepsine was applied for 6 min at RT and the washing step was repeated. After dehydration in graded ethanol slides were left to dry at RT for 6 min, and after addition of 10 μl HER2/Chr17 probe mix a coverslip with sealant was applied and slides were placed in the ThermoBrite Hybridizer (StatSpin, Norwood, MA, USA). Denaturation step was 5 min at 82°C followed by overnight hybridization at 45°C. Slides were then retrieved from the Hybridizer, coverslips were removed and stringent washing at 65°C was carried out followed by wash buffer treatment. Finally, mounting medium was applied with a new coverslip. Slides were stored at −20°C until assessment.

ISH assessment was performed on a Zeiss Axioscope 40 fluorescence microscope with ×40/N.A.1.3 and ×100/N.A.1.3 (oil) objectives. SISH slides were counted using the same objectives but with bright field light. Before ISH assessment the serial slides stained with H&E, SP3 and 4B5 antibodies were re-evaluated. In the ISH slides, all tumour areas were assessed. Areas containing the highest HER2 counts were assessed preferentially by counting HER2 and Chr17 signals in at least 20 nuclei. HER2:Chr17 ratios were calculated. When counts exceeded 300 per 20 nuclei, >300 was noted, and a HER2:Chr17 ratio of >6 was assumed. Positive ISH was defined as a HER2:Chr17 ratio of ≥2.2. Polysomy was defined as an average of ≥3 Chr17 signals per nucleus. Of all slides with dual (SISH and FISH) assessments, photomicrographs were taken and stored in Research Assistant 5 (RVC, Baarn, the Netherlands).

## Results

### Tumour Characteristics

Histological characterics and the anatomical location of the 146 gastro-oesophageal tumours are presented in Table 1. Biopsies of 72 adenocarcinomas were from the EGR,; 44 (61%) from the distal oesophagus and 28 (39%) from the gastric cardia. Sixty-three were classified as intestinal type, six as mixed and three as mucinous. Seventy-four adenocarcinomas were from the distal stomach, 24 (32%) in the gastric body and 50 (68%) in the antrum. Fifty-four were of intestinal type, nine mixed, nine diffuse and two of mucinous type (Table 1).

**Table 1:** Histological^37^ classification and anatomical position of the adenocarcinomas

	Anatomical position					
	
Laurén classification	Oesophagus	Cardia	Total EGR	Body	Antrum	Total distal stomach
Intestinal	40 (91%)	23 (82%)	63 (88%)	15 (62%)	39 (78%)	54 (73%)

Mixed	3 (7%)	3 (11%)	6 (8%)	6 (25%)	3 (6%)	9 (12%)

Diffuse	1 (2%)	2 (7%)	3 (4%)	3 (13%)	6 (12%)	9 (12%)

Mucinous	0 (0%)	0 (0%)	0 (0%)	0 (0%)	2 (4%)	2 (3%)

Total	44 (100%)	28 (100%)	72 (100%)	24 (100%)	50 (100%)	74 (100%)

EGR, Oesophago-gastric region.

### HER2 Immunohistochemistry

**Figures 1 and 2:**
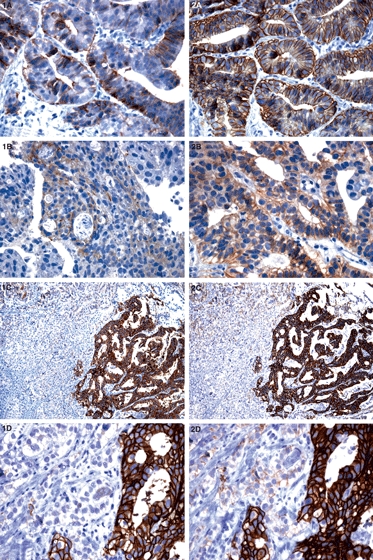
Figures 1 [SP3 immunohistochemistry (IHC)] and 2 (4B5 IHC) show identical cases. **1A**, basolateral staining in SP3 (score 1+) but in **2A** a 3+ staining pattern in 4B5; **1B**, **2B**, 1+ staining in both SP3 and 4B5; **1C**, **2C** (low magnification)**,** heterogeneous staining with 3+ pattern in the right half of the tumour and a 1+ staining in the left side, identical in SP3 and 4B5; **1D**, **2D**, detail of the 1+/3+ transition zone.

**Figure 5:**
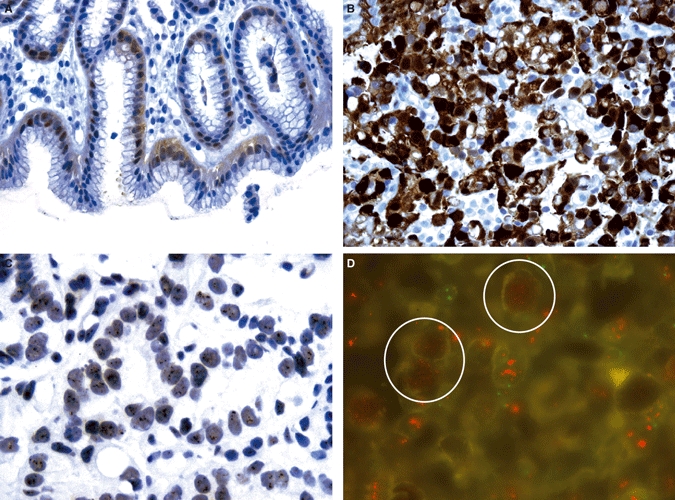
**A**, 4B5 immunohistochemistry (IHC): background staining in foveolar layer of gastric epithelium; **B**, 4B5 IHC: strong nuclear and cytoplasmic background staining in a diffuse type adenocarcinoma; **C**, human epidermal growth factor receptor 2 (HER2) silver *in situ* hybridization (SISH): nuclear haze; **D**, fluorescence *in situ* hybridization (FISH): red granular background staining of some nuclei (encircled) among nuclei showing high amplification.

SP3 immunoreactivity (Figure 1) was completely absent in 125 (86%) cases. An immunoscore of 1+ was seen in four, 2+ in six and 3+ in 11 cases. Membranous 4B5 immunoreactivity (Figure 2) was absent in 106 (72%) cases; immunoscores of 1+ were present in 17 cases, 2+ in six and 3+ in 17. All cases with immunoscores of 1+ and higher showed immunoreactivity in at least 10% of tumour cells. *U*-shaped basolateral staining was often noted. Background staining was entirely absent with the SP3-antibody. The 4B5 stains showed invariably extensive cytoplasmic background staining of the gastric foveolar layer (Figure 5A); intestinal metaplasia when present also showed this staining pattern, but was less substantial. In addition, cytoplasmic background staining of tumour was noted in 40 (27%) cases, of which seven (5%) were strong with nuclear immunoreactivity. In an additional two cases, only strong background staining was noted without notable membranous staining (Figure 5B) (Table 2).

**Table 2:** SP3 versus 4B5 human epidermal growth factor receptor 2 (HER2) immunoscores

	4B5				
	
SP3	0	1+	2+	3+	Total
0	105	15	5	0	125 (86%)

1+	1	2	1	0	4 (3%)

2+	0	0	0	6	6 (4%)

3+	0	0	0	11	11 (7%)

Total	106 (72%)	17 (12%)	6 (4%)	17 (12%)	146 (100%)

### HER2 SISH/FISH *Versus* Immunoscore

**Figures 3 and 4:**
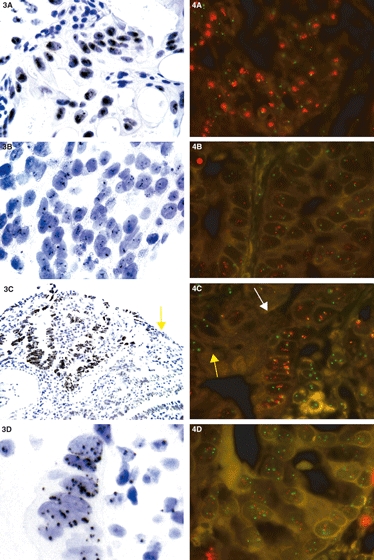
Figures 3 [silver *in situ* hybridization (SISH) and 4 [fluorescence *in situ* hybridization (FISH)] with identical cases. **3A**, **4A**, high amplification; **3B**, **4B**, low amplification; **3C**, **4C** (lower magnification)**,** area with (white arrow) and without (yellow arrow) magnification; **3D**, chromosome 17 SISH: polysomy with >3 copies chromosome 17 per nucleus; **4D**, polysomy. FISH: red dots, human epidermal growth factor receptor 2 (HER2); green dots, chromosome 17.

SISH was performed in all 146 cases (Table 3a; Figure 3). In some cases of SISH some nuclear haze was present, possibly as a result of the fixation time of approximately 6 h (Figure 5C). All 17 cases with SP3 immunoscores 2+/3+ were amplified, as were five of 129 (4%) cases with negative SP3 immunohistochemistry (immunoscores 0/1+). Only one case of 123 with 4B5 immunoscores 0/1+ was amplified, and 21 of 23 (91%) cases with immunoscores 2+/3+. The two amplified cases with SP3-immunoscore 0 were scored 2+ on 4B5 immunohistochemistry. The three amplified cases with SP3-immunoscore 1+ were 2+ in the 4B5 in two cases and 1+ in one case. Two cases with strong 4B5 background staining of nuclei (but no membranous staining) were not amplified.

**Table 3:** Human epidermal growth factor receptor 2 (HER2) SP3 and 4B5 immunoscores versus (a) silver *in situ* hybridization (SISH), (b) fluorescence *in situ* hybridization (FISH)

	ISH				ISH		
			
SP3	Negative	Positive	Total	4B5	Negative	Positive	Total
(a)							
0	123 (98%)	2 (2%)	125	0	106 (100%)	0 (0%)	106

1+	1 (25%)	3 (75%)	4	1+	16 (94%)	1 (6%)	17

2+	0 (0%)	6 (100%)	6	2+	2 (33%)	4 (66%)	6

3+	0 (0%)	11 (100%)	11	3+	0 (0%)	17 (100%)	17

Total	124	22	146	Total	124	22	146

(b)							
0	19 (90%)	2 (10%)	21	0	2 (100%)	0 (0%)	2

1+	1 (25%)	3 (75%)	4	1+	16 (94%)	1 (6%)	17

2+	0 (0%)	6 (100%)	6	2+	2 (33%)	4 (66%)	6

3+	0 (0%)	11 (100%)	11	3+	0 (0%)	17 (100%)	17

Total	20	22	42	Total	20	22	42

Sensitivity for amplification was 77.3% for SP3 and 95.5% 4B5 IHC-positive cases as defined by immunoscores 2+/3+. Specificity was 100% for SP3-positive IHC and 98.4% for 4B5-positive IHC, respectively. The positive predictive value (PPV) of SP3-positivity was 100% and the negative predictive value (NPV) 96.1%. For 4B5 the PPV was 91.3% and the NPV 99.2%.

Of 42 FISH procedures performed (Figure 4) the results were identical to SISH. Twenty-two cases were amplified (Table 3b). Granular red background staining was seen in a minority of cases in both tumour and normal epithelial cells (Figure 5D).

### Heterogeneity of HER2 Positivity

Heterogeneity of HER2-immunoreactivity was the dominant pattern, and areas of HER2 amplification closely matched positive HER2-immunoreactivity. The percentage of tumour staining for an antibody without regard to immunoscore, the number of positive biopsies and/or calculated H-score did not predict amplification status. The only generalization that could be made was that most amplified cases had a 2+/3+ immunoscore with SP3, and 3+ (any percentage) or 2+ (>50% tumour area) with 4B5 IHC (Table 4).

**Table 4:** SP3 and 4B5 immunoscores versus percentage (IHC)-positive cells in amplified/non-amplified tumours

ISH negative								ISH positive					
		
	% positive cells in IHC							% positive cells in IHC					
					
SP3	0	1–10	11–50	51–75	76–100	Subtotal	SP3	0	1–10	11–50	51–75	76–100	Subtotal
0	123	0	0	0	0	123	0	0	1	1	0	0	2

1+	0	0	0	0	1	1	1+	0	0	2	0	1	3

2+	0	0	0	0	0	0	2+	0	0	2	0	4	6

3+	0	0	0	0	0	0	3+	0	0	4	0	7	11

4B5	0	1–10	11–50	51–75	76–100	Subtotal	4B5	0	1–10	11–50	51–75	76–100	Subtotal

0	106	0	0	0	0	106	0	0	0	0	0	0	0

1+	0	3	10	0	3	16	1+	0	0	1	0	0	1

2+	0	0	2	0	0	2	2+	0	1	2	0	1	4

3+	0	0	0	0	0	0	3+	0	0	6	0	11	17

ISH, *In situ* hybridization.

### Polysomy

Polysomy defined as ≥3 Chr17 copies per cell was present in 28 cases (19.2%). Polysomy was noted in seven of 22 (32%) amplified cases showing invariably ≥5 HER2 signals per nucleus. In contrast, polysomy present in 21 of 104 cases had <5 HER2 signals per nucleus. Therefore, assessment of polysomy – often a striking finding in tumours in our study – did not contribute to the prediction of amplification.

### HER2 Amplification and Anatomical Location/Histological Type

Of the total of 117 adenocarcinomas of intestinal type, 20 (17%) showed HER2-amplification; 17 cases were located in the EGR and two in the distal stomach. Two of 15 cases (13%) with mixed-type histology were also amplified and were located in the distal stomach. None of the 14 cases with diffuse or mucinous types showed amplification (Table 5).

**Table 5:** Number (and percentage of biopsies) of human epidermal growth factor receptor 2 (HER2) amplified cases versus Laurén classification and anatomical location

	Anatomical position					
	
Laurén	Oesophagus	Cardia	Total EGR	Body	Antrum	Total distal stomach
Intestinal	12 (30%)	5 (22%)	17 (27%)	1 (7%)	2 (5%)	3 (6%)

Mixed	0 (0%)	0 (0%)	0 (0%)	1 (17%)	1 (33%)	2 (22%)

Diffuse	0 (0%)	0 (0%)	0 (0%)	0 (0%)	0 (0%)	0 (0%)

Mucinous	0 (0%)	0 (0%)	0 (0%)	0 (0%)	0 (0%)	0 (0%)

Total	12 (27%)	5 (18%)	17 (24%)	2 (8%)	3 (6%)	5 (7%)

EGR, Oesophago-gastric region.

Of the tumours situated in the EGR, 17 (24%) were HER2 amplified: 12 of 44 distal oesophageal adenocarcinomas and five of 28 cardia carcinomas. In the distal stomach, five (7%) showed HER2 amplification: two of 24 body and three of antral carcinomas.

## Discussion

In a study of 146 cases with biopsies of primary oesophagus and gastric adenocarcinomas, frequent HER2-immunoreactivity using two novel rabbit monoclonal antibodies was identified. Both SP3 and 4B5 immunostaining had a high positive predictive value for HER2-amplification. 4B5 had a much higher negative predictive value than SP3. SISH, when compared with FISH in selected cases, yielded identical results compared with FISH. HER2-amplification was most often present in biopsies of oesophagus and gastric cardia adenocarcinomas (27% and 18%, respectively); amplification was less frequent in adenocarcinomas of the body and antral region (7%).

Many studies have been published on the concordance of HER2 immunohistochemistry and *in situ* hybridization in breast cancer,^25^ focusing usually upon the rabbit polyclonal antibody A408 (used in HercepTest™) or the mouse monoclonal CB11 (used in the previous generation of Ventana Pathway®; Ventana), both FDA-approved kits. In daily practice the application of these antibodies results in a relatively high percentage of equivocal 2+ immunoscores. In order to achieve an unequivocal 3+ immunoscore pattern in cases with high amplification a high background in normal tissue has to be accepted, precluding easy and reproducible assessment of immunohistochemistry. The new generation of more avid rabbit monoclonal antibodies has been developed in order to produce better signal-to-background patterns. In the field of oestrogen receptor immunohistochemistry, the new SP1-antibody has become an acclaimed test. SP3 and 4B5 are new rabbit monoclonal antibodies directed against the intracellular and extracellular of the HER2-receptor, respectively.

Ricardo *et al.*^28^ reported a significant level of agreement between SP3 and CB11 IHC in breast carcinomas and chromogenic *in situ* hybridization (CISH), and claimed easier assessment of SP3 immunostains. However, of 50 CISH-amplified samples 21 cases showed negative SP3/CB11 IHC scores. Nunes *et al.*^31^ reported a better negative predictive value of SP3 immunohistochemistry, with CB11 being slightly inferior, but the Herceptest being equivalent to SP3. In a recent update of UK recommendations on HER2 testing,^32^ Walker *et al.* reported equivalent results of 4B5 in comparison with Herceptest, which is in concordance with a report of the quality assurance programme Nordiqc.^33^

In our study, both the SP3 and 4B5 yielded good correlations with both *in situ* hybridization methods. SP3 IHC produced clear immunostains facilitating rapid and easy assessment. However, five of the 22 amplified cases were SP3-IHC negative, two (of 21) cases with a complete negative staining pattern and three (of four) cases with a 1+ pattern. The 4B5 antibody exhibited different staining characteristics: only one (of 19) negative in IHC (1+ score) case was amplified, as were 20 (of 22) cases with positive IHC (score 2+/3+). The 4B5 was considerably more sensitive than the SP3, with a better correlation with *in situ* hybridization. However, some drawbacks of the 4B5 IHC were apparent: distinct cytoplasmic background staining was present almost invariably in the foveolar layer of the stomach; in addition, strong cytoplasmic and even nuclear background could be seen in some tumour areas. No amplification was present in the foveolar or tumour cells exhibiting background staining but assessment of IHC was occasionally hindered by these phenomena, which are not seen in breast pathology.

Due to its recent introduction, few studies comparing 4B5 with other antibodies have been published in breast carcinomas. Powell *et al.*^33^ from Ventana reported sharper membrane staining, less background and better concordance with FISH using 4B5 in 322 breast carcinomas in comparison with CB11. Egervari *et al.*^34^ compared six HER2 antibodies in a tissue microarray (TMA) composed of 199 breast cancers with FISH. In this array, amplification was present in 23 cases. In contrast to our findings, they found that some cases with immunoscores of 0/1+ with 4B5 IHC showed amplification. This underscores the need for in-house validation of immunohistochemistry prior to limiting *in situ* hybridization to the category of 2+ immunoscores, as required by 2007 ASCO/CAP HER2 guidelines for breast cancer.^25^

Reported frequencies of positive HER2-immunoreactivity in gastro-oesophageal cancer vary extensively. Table 6 lists the studies with full-paper reports in which *in situ* hybridization has been carried out in more than 50 cases. A comprehensive review by Hofmann *et al.*^3^ described 3264 cases of gastric adenocarcinoma showing a positivity rate of 17.6% defined by IHC. In our selection of ISH-confirmed HER2 status in Table 6, this percentage is lowered to 12%. Even after detailed review of the full-paper reports, it remains unclear whether adenocarcinomas of the gastric cardia are included in the studies listed in Table 6, let alone the relative contribution of these tumours to overall findings. Thus, our finding of a 7% HER2-positivity rate in adenocarcinomas of the distal stomach is no outlier; if cardia carcinomas are included, an 11% positivity rate is reached in the present study. Bang *et al.*^2^ claimed a 22.1% HER2-positivity in 3280 cases of advanced, recurrent and/or metastatic gastric adenocarcinomas, as defined by either 3+ immunoscore using HercepTest™ or amplification with Dako FISH in an abstracted ToGA trial report. Adenocarcinoma of the stomach and the gastro–oesophageal junction had a HER2-positivity of 20.9% and 33.2%, respectively. This is substantially higher than most reports, including the present study. In contrast to the present study, they found 4.9% FISH positive cases in which IHC was completely negative, and a 15% amplification rate when the immunoscore was 1+. Subsequent analysis failed to show a clinical benefit of trastuzumab in the FISH-positive but immunonegative subgroups. Another possible explanation might be that patients with advanced disease only were accrued for the ToGA trial, while HER2 amplification could be seen to be a late event in oncogenesis. The average HER2-positivity rates of the patients included in the ToGA at the time of the report by Bang *et al.* did not vary according to the geographical region (Europe or Asia), but was a function of histological type in concordance with our study.

**Table 6:** Human epidermal growth factor receptor 2 (HER2) status in studies with *in situ* hybridization (ISH) on adenocarcinomas

Reference	Method	*n* (Total)	*n* (Positive)	% (Positive)
Stomach				
Ishikawa^36^	FISH	105	19	18%

Takehana^14^	FISH	352	29	8%

Risio^18^	FISH	72	11	15%

Varis^37^	CISH	52	9	17%

Tanner^11^	CISH	131	16	12%

Park^9^	FISH/CISH	182	7	4%

Yano^38^	FISH	200	54	27%

Kim^8^	FISH	248	19	8%

Barros-Silva^19^	FISH	463	38	8%

Marx^1^	FISH	166	27	16%

Total		1.981	229	Mean 12%

Oesophagus				
Brien^16^	FISH	63	12	19%

Tanner^11^	CISH	100	24	24%

Reichelt^4^	FISH	110	16	15%

Total		273	52	Mean 19%

FISH, Fluorescence *in situ* hybridization; CISH, chromogenic *in situ* hybridization.

The few larger studies with *in situ* hybridization-confirmed HER2 status in oesophageal adenocarcinoma report a HER2-positivity rate of at least 15%. Our 27% HER2 amplification rate is in line with substantial higher HER2-positivity in oesophageal carcinoma rather than (distal) gastric adenocarcinoma. Unfortunately, questions concerning definitions, segmentation of oncological entities, division of treatment groups and different research interests hinder a comprehensive view of HER2-positivity rates of the anatomical regions comprising the distal oesophagus and the entire stomach.

An important difference in HER2 immunoreactivity and amplification between breast cancer and gastro-oesophageal adenocarcinomas is the striking heterogeneity of HER2-positivity in the latter. The 2007 ASCO/CAP HER2 Guidelines^25^ in breast cancer demands HER2-immunoreactivity/amplification in at least 30% of breast cancer tumour areas in order to render a HER2-positivity as a conclusion. Hofmann *et al.*^3^ has proposed allowing even less than the original HercepTest™ 10% 3+ pattern of immunoreactivity in biopsies of gastric carcinomas; in resection specimens the 10% threshold is retained. The considerable heterogeneity of HER2-positivity – 73% of all HER2-amplified cases in our study – suggests that HER2 amplification is a late event in gastro-oesophageal adenocarcinomas. Nevertheless, a recent report of a large Phase III trial using trastuzumab (ToGA trial^20^) showed clinical benefit, especially in the group with both FISH positivity and an immunoscore of at least 2+ using Herceptest. The magnitude of clinical benefit is similar to metastatic breast carcinomas. This is remarkable, considering the heterogeneous HER2-positivity in gastric adenocarcinomas in contrast to breast cancer, in which more than 95% of HER2-positive cases are HER2-amplified homogeneously. Recently, Marx *et al.*^1^ reported highly homogeneous HER2 amplification in multiple sections obtained from eight highly amplified cancers. It is possible that this is a selection phenomenon, as they studied 166 surgically resected gastric cancers cases, 27 of which showed unequivocal amplification.

Silver *in situ* hybridization is a recently introduced technique, being somewhat similar to CISH in that a conventional microscope can be used to assess the probe signals. Reports are still limited,^29^,^35^ with fewer than 150 cases examined; so far, good correlations between FISH and SISH have been described. In our study, 42 cases yielded identical results using both techniques. Assessment of HER2/Chr17 signal in the multiple biopsies was much faster and easier with SISH in comparison with FISH, and allows a good correlation between IHC and SISH using conventional techniques such as ink-marking.

Most contemporary studies outside trials use the very cost-effective TMA technique. However, given the high incidence of heterogeneous HER2-immunoreactivity/amplification, we felt that using the TMA technique in this field could result in an underestimation of the incidence of HER2-amplification rates. While we did not study resection specimens, the claim by Marx *et al.*^1^ that HER2 amplification is highly homogeneous is contradicted by our findings in biopsy material.

## Abbreviations

ASCO: American Society of Clinical Oncology
CAP: College of American Pathologists
Chr17: chromosome 17
CISH: chromogen *in situ* hybridization
DAB: 3,3′ dia minobenzidine
EGJ: oesophago-gastric junction
EGR: oesophago-gastric region
FDA: Federal Drug Agency
FISH: fluorescence *in situ* hybridization
H&E: haematoxylin and eosin
HER1: human epidermal growth factor receptor 1
HER2: human epidermal growth factor receptor 2
H-score: histoscore
IHC: immunohistochemistry
ISH: *in situ* hybridization
NA: numerical aperture
NPV: negative predictive value
PPV: positive predictive value
RT: room temperature
SISH: silver *in situ* hybridization
TMA: tissue microarray

## References

[1] Marx AH, Tharun L, Muth J (2009). HER-2 amplification is highly homogenous in gastric cancer. Hum. Pathol..

[2] Bang YJ, Chung HC, Sawaki S (2008). HER2-positivity rates in advanced gastric cancer: results from a large international phase III trial. J. Clin. Oncol..

[3] Hofmann M, Stoss O, Shi D (2008). Assessment of a HER2 scoring system for gastric cancer: results from a validation study. Histopathology.

[4] Reichelt U, Duesedau P, Tsourlakis M (2007). Frequent homogeneous HER-2 amplification in primary and metastatic adenocarcinoma of the esophagus. Mod. Pathol..

[5] Ross JS, McKenna BJ (2001). The HER-2/neu oncogene in tumors of the gastrointestinal tract. Cancer Invest..

[6] Tapia C, Glatz K, Novotny H (2007). Close association between HER-2 amplification and overexpression in human tumors of non-breast origin. Mod. Pathol..

[7] Kanta SY, Yamane T, Dobashi Y, Mitsui F, Kono K, Ooi A (2006). Topoisomerase IIalpha gene amplification in gastric carcinomas: correlation with the HER2 gene. An immunohistochemical, immunoblotting, and multicolor fluorescence in situ hybridization study. Hum. Pathol..

[8] Kim MA, Jung EJ, Lee HS (2007). Evaluation of HER-2 gene status in gastric carcinoma using immunohistochemistry, fluorescence *in situ* hybridization, and real-time quantitative polymerase chain reaction. Hum. Pathol..

[9] Park DI, Yun JW, Park JH (2006). HER-2/neu amplification is an independent prognostic factor in gastric cancer. Dig. Dis. Sci..

[10] Miller CT, Moy JR, Lin L (2003). Gene amplification in esophageal adenocarcinomas and Barrett's with high-grade dysplasia. Clin. Cancer Res..

[11] Tanner M, Hollmen M, Junttila TT (2005). Amplification of HER-2 in gastric carcinoma: association with Topoisomerase IIalpha gene amplification, intestinal type, poor prognosis and sensitivity to trastuzumab. Ann. Oncol..

[12] Flejou JF, Paraf F, Muzeau F (1994). Expression of c-erbB-2 oncogene product in Barrett's adenocarcinoma: pathological and prognostic correlations. J. Clin. Pathol..

[13] Tsugawa K, Fushida S, Yonemura Y (1993). Amplification of the c-erbB-2 gene in gastric carcinoma: correlation with survival. Oncology.

[14] Takehana T, Kunitomo K, Kono K (2002). Status of c-erbB-2 in gastric adenocarcinoma: a comparative study of immunohistochemistry, fluorescence *in situ* hybridization and enzyme-linked immuno-sorbent assay. Int. J. Cancer.

[15] Motojima K, Furui J, Kohara N, Izawa K, Kanematsu T, Shiku H (1994). erbB-2 expression in well-differentiated adenocarcinoma of the stomach predicts shorter survival after curative resection. Surgery.

[16] Brien TP, Odze RD, Sheehan CE, McKenna BJ, Ross JS (2000). HER-2/neu gene amplification by FISH predicts poor survival in Barrett's esophagus-associated adenocarcinoma. Hum. Pathol..

[17] Jaehne J, Urmacher C, Thaler HT, Friedlander-Klar H, Cordon-Cardo C, Meyer HJ (1992). Expression of Her2/neu oncogene product p185 in correlation to clinicopathological and prognostic factors of gastric carcinoma. J. Cancer Res. Clin. Oncol..

[18] Risio M, De Rosa G, Sarotto I (2003). HER2 testing in gastric cancer: molecular morphology and storage time-related changes in archival samples. Int. J. Oncol..

[19] Barros-Silva JD, Leitao D, Afonso L (2009). Association of ERBB2 gene status with histopathological parameters and disease-specific survival in gastric carcinoma patients. Br. J. Cancer.

[20] Bang YJ, Van Cutsem E, Feyereislova A, Chung HC, Shen L, Sawaki A, Lordick F, Ohtsu A, Omuro Y, Satoh T, Aprile G, Kulikov E, Hill J, Lehle M, Ruschoff J, Kang YK (2010). Trastuzumab in combination with chemotherapy versus chemotherapy alone for treatment of HER2-positive advanced gastric or gastro-oesophageal junction cancer (ToGA): a phase 3, open-label, randomised controlled trial. Lancet.

[21] Tew WP, Kelsen DP, Ilson DH (2005). Targeted therapies for esophageal cancer. Oncologist.

[22] Hamilton SR, Aaltonen LA (2000). Pathology and genetics of tumours of the digestive system.

[23] Enzinger PC, Mayer RJ (2003). Esophageal cancer. N. Engl. J. Med..

[24] Dicken BJ, Bigam DL, Cass C, Mackey JR, Joy AA, Hamilton SM (2005). Gastric adenocarcinoma: review and considerations for future directions. Ann. Surg..

[25] Wolff AC, Hammond ME, Schwartz JN (2007). American Society of Clinical Oncology/College of American Pathologists guideline recommendations for human epidermal growth factor receptor 2 testing in breast cancer. Arch. Pathol. Lab. Med..

[26] Milanezi F, Carvalho S, Schmitt FC (2008). EGFR/HER2 in breast cancer: a biological approach for molecular diagnosis and therapy. Expert Rev. Mol. Diagn..

[27] Sheppard B (2007). Results of the College of American Pathologists HER2 proficiency exam.

[28] Ricardo SA, Milanezi F, Carvalho ST, Leitao DR, Schmitt FC (2007). HER2 evaluation using the novel rabbit monoclonal antibody SP3 and CISH in tissue microarrays of invasive breast carcinomas. J. Clin. Pathol..

[29] Dietel M, Ellis IO, Hofler H (2007). Comparison of automated silver enhanced in situ hybridisation (SISH) and fluorescence ISH (FISH) for the validation of HER2 gene status in breast carcinoma according to the guidelines of the American Society of Clinical Oncology and the College of American Pathologists. Virchows Arch..

[30] Nunes CB, Rocha RM, Reis-Filho JS (2008). Comparative analysis of six different antibodies against Her2 including the novel rabbit monoclonal antibody (SP3) and chromogenic *in situ* hybridisation in breast carcinomas. J. Clin. Pathol..

[31] Walker RA, Bartlett JM, Dowsett M (2008). HER2 testing in the UK: further update to recommendations. J. Clin. Pathol..

[32] Nordiqc Assessment Run B6 2008. http://www.nordiqc.org/Run-24-B6/Assessment/assessment-HER-2.htm.

[33] Powell WC, Hicks DG, Prescott N (2007). A new rabbit monoclonal antibody (4B5) for the immunohistochemical (IHC) determination of the HER2 status in breast cancer: comparison with CB11, fluorescence *in situ* hybridization (FISH), and interlaboratory reproducibility. Appl. Immunohistochem. Mol. Morphol..

[34] Egervari K, Szollosi Z, Nemes Z (2008). Immunohistochemical antibodies in breast cancer HER2 diagnostics. A comparative immunohistochemical and fluorescence in situ hybridization study. Tumour Biol..

[35] Capizzi E, Gruppioni E, Grigioni AD (2008). Real time RT–PCR approach for the evaluation of ERBB2 overexpression in breast cancer archival samples: a comparative study with FISH, SISH, and immunohistochemistry. Diagn. Mol. Pathol..

[36] Ishikawa T, Kobayashi M, Mai M, Suzuki T, Ooi A (1997). Amplification of the c-erbB-2 (HER-2/neu) gene in gastric cancer cells. Detection by fluorescence in situ hybridization. Am. J. Pathol..

[37] Varis A, Zaika A, Puolakkainen P (2004). Coamplified and overexpressed genes at ERBB2 locus in gastric cancer. Int. J. Cancer.

[38] Yano T, Doi T, Ohtsu A (2006). Comparison of HER2 gene amplification assessed by fluorescence *in situ* hybridization and HER2 protein expression assessed by immunohistochemistry in gastric cancer. Oncol. Rep..

